# Optimized approach for the identification of highly efficient correctors of nonsense mutations in human diseases

**DOI:** 10.1371/journal.pone.0187930

**Published:** 2017-11-13

**Authors:** Hana Benhabiles, Sara Gonzalez-Hilarion, Séverine Amand, Christine Bailly, Anne Prévotat, Philippe Reix, Dominique Hubert, Eric Adriaenssens, Sylvie Rebuffat, David Tulasne, Fabrice Lejeune

**Affiliations:** 1 Univ. Lille, CNRS, Institut Pasteur de Lille, UMR 8161—M3T –Mechanisms of Tumorigenesis and Target Therapies, Lille, France; 2 Muséum National d'Histoire Naturelle, Sorbonne Universités, Centre National de la Recherche Scientifique, Laboratoire Molécules de Communication et Adaptation des Microorganismes (MCAM), UMR 7245 CNRS-MNHN, CP 54, Paris, France; 3 Univ. Lille, Clinique des Maladies Respiratoires, CRCM Hôpital Calmette, CHRU Lille, France; 4 Hospices Civils de Lyon, Centre de Référence Mucoviscidose, Lyon, France; 5 Pulmonary Department and Adult CF Centre, Cochin Hospital, AP-HP, Paris, France; 6 INSERM U908, Cell plasticity and Cancer, University of Lille, Villeneuve d'Ascq, France; Medical Faculty Mannheim, University of Heidelberg, GERMANY

## Abstract

About 10% of patients with a genetic disease carry a nonsense mutation causing their pathology. A strategy for correcting nonsense mutations is premature termination codon (PTC) readthrough, i.e. incorporation of an amino acid at the PTC position during translation. PTC-readthrough-activating molecules appear as promising therapeutic tools for these patients. Unfortunately, the molecules shown to induce PTC readthrough show low efficacy, probably because the mRNAs carrying a nonsense mutation are scarce, as they are also substrates of the quality control mechanism called nonsense-mediated mRNA decay (NMD). The screening systems previously developed to identify readthrough-promoting molecules used cDNA constructs encoding mRNAs immune to NMD. As the molecules identified were not selected for the ability to correct nonsense mutations on NMD-prone PTC-mRNAs, they could be unsuitable for the context of nonsense-mutation-linked human pathologies. Here, a screening system based on an NMD-prone mRNA is described. It should be suitable for identifying molecules capable of efficiently rescuing the expression of human genes harboring a nonsense mutation. This system should favor the discovery of candidate drugs for treating genetic diseases caused by nonsense mutations. One hit selected with this screening system is presented and validated on cells from three cystic fibrosis patients.

## Introduction

A nonsense mutation changes a codon into a UAA, UAG, or UGA stop codon. Instead of causing synthesis of a truncated protein, the presence of a premature termination codon (PTC) in an mRNA promotes silencing of the mutant gene when the PTC position fits some specific rules, due to rapid decay of the nonsense-mutation-containing mRNA by a mechanism called nonsense-mediated mRNA decay (NMD) [1,2,3,4,5,6,7,8]. In yeast, NMD is activated according to the length of the 3’ untranslated region (3’UTR) [9]. When the 3’UTR appears abnormally long, and thus notably in the presence of a PTC, the mRNA is targeted for NMD [9]. In human, activation of NMD depends on the relative position of the first stop codon of the open reading frame (ORF) with respect to the positions of downstream splicing events. If the first stop codon on an mRNA is located more than 50–55 nucleotides upstream of an exon-exon junction, NMD will be elicited on that mRNA [10]. A second pathway of NMD activation has been described in human cells, involving the distance between the poly(A) binding protein C1 (PABPC1) and the first stop codon of an ORF. According to this model, if the distance between the first stop codon and the PABPC1 is recognized as abnormally long, as when a PTC is present, NMD will be elicited [1,11,12,13,14]. Since experimental arguments exist in support of both activation pathways, NMD in human cells might simply be activated by either of these pathways, according to model which includes them both [15,16].

Nonsense mutations can cause rare genetic diseases such as Duchenne muscular dystrophy, cystic fibrosis, and hemophilia, and also frequent diseases such as cancers, metabolic disorders, and neurological disorders [16,17]. Several strategies have been proposed to correct nonsense mutations. One is to favor PTC readthrough, a process in which an amino acid is incorporated into the nascent polypeptide chain when the ribosome is at the PTC position, so as to complete translation of the ORF [16,18,19,20,21,22]. Correcting a nonsense mutation rescues the functional expression of the gene carrying that mutation. PTC readthrough results in synthesis of a full-length protein that might differ by one amino acid from the wild-type protein, since the amino acid incorporated at the PTC position can be different from that encoded by the wild-type DNA sequence. The readthrough protein will be functional unless the amino acid introduced at the PTC position is not compatible with the function of the protein. In human cells, readthrough has recently been shown to occur in specific cytoplasmic foci called readthrough bodies and requires the NMD factors UPF1, UPF2, and UPF3X [23].

The efficiency of readthrough is limited. One reason for this is that PTC-mRNAs are targeted by NMD before they can serve as substrates for PTC readthrough. Nevertheless, up to 25% of PTC-mRNAs escape NMD and can become substrates for PTC readthrough [24]. In addition, the readthrough efficiency depends on the identity of the stop codon to be read through. On the basis of experiments using readthrough molecules of the aminoglycoside family (gentamicin, geneticin (G418), tobramycin, or amikacin, for example), the UGA stop codon has been ranked as the most permissive and the UAA codon as the least permissive for readthrough [25]. The nucleotide context surrounding the PTC also influences the efficiency of readthrough. The best nucleotide environment for eliciting readthrough of a UAA or UGA stop codon appears to be a U before the stop codon and a "CUAG" sequence after it. The environment most favorable to readthrough of a UAG stop codon appears to be a U before the stop codon and a “UUAG” after it [26,27].

Although aminoglycosides show the capacity to elicit PTC readthrough, the clinical development of aminoglycosides was stopped because of evidence of irreversible ototoxicity and reversible nephrotoxicity after long exposures [28,29,30,31,32,33]. Since then, less toxic aminoglycoside derivatives and non-aminoglycoside molecules have been tested. The molecules shown to induce PTC readthrough notably include ataluren (formerly called PTC124), RTC compounds, and amlexanox [34,35,36,37,38,39,40,41,42]. Ataluren does not seem to cause toxicity, and clinical trials have shown it to be very well tolerated by patients [43,44]. Although the efficacy of ataluren is controversial, some benefits have been reported, supporting interest in correcting nonsense mutations as a treatment for human genetic pathologies [43,44,45,46,47,48,49,50]. The screening systems developed to identify ataluren and other readthrough compounds measured an enzymatic activity or a fluorescence associated with a construct bearing a nonsense-mutation-containing cDNA [34,51]. The encoded mRNAs escape NMD because they undergo no splicing downstream of the PTC. This situation contrasts with the natural environment occurring in patient cells, since in human cells about 97% of human genes express pre-mRNAs subject to splicing [52,53,54]. Use of an unspliced reporter might explain the low efficacy of the molecules identified to date when tested on human cells [55], since spliced PTC-mRNAs are subject to NMD, which strongly reduces the amount of PTC-mRNA available for readthrough [56]. To improve the PTC readthrough efficiency, it has been proposed to combine NMD inhibition (so as to increase the level of PTC-mRNAs) with activation of PTC readthrough [42,57,58]. When the aim is to propose new drug candidates capable of effectively correcting nonsense mutations in human cells, one should reproduce with the reporter gene the situation of a nonsense mutation in a gene containing exonic and intronic sequences. We have thus designed and built a screening system dedicated to the identification of compounds capable of rescuing, in human cells, the expression of a gene harboring a nonsense mutation. This screening system uses a reporter mRNA subject to NMD because of a splicing event occurring downstream of a PTC. The selected compounds will thus be able to correct nonsense mutations on spliced mRNAs, either by inhibiting NMD and activating readthrough or by very strongly activating readthrough on the residual fraction of PTC-mRNAs having escaped NMD. We have tested the system with a known nonsense mutation corrector to establish proof of concept and have used it to screen a library of marine invertebrate and fungal extracts. We present the identification and validation of an extract capable of rescuing very effectively the expression of human genes harboring a UGA or a UAA nonsense mutation. The results of the screen are supported by a straightforward and relevant validation procedure applied to several immortalized cell lines with different endogenous nonsense mutations and to cystic fibrosis patient cells.

## Materials and methods

### Constructs

The firefly luciferase gene used for screening was constructed by first introducing an intron into the cDNA sequence of firefly luciferase as previously described [59]. Briefly, a 132-bp chimeric βglobin/immunoglobulin intron sequence (Promega, Charbonnières-les-Bains, France) was introduced at position 1344 bp of the firefly luciferase gene. A nonsense mutation (TGA, TAG, or TAA) was then introduced at the position corresponding to codon 109, with the QuickChange Site-Directed Mutagenesis kit (ThermoFisher Scientific, Waltham, MA, USA) used according to the manufacturer’s recommendations.

### The luciferase construct under a p53-responsive promoter

The primers (P53 sense: 5'CAGACATGCCTAGACATGCCTAACCGGTTAGACATGCCTAGACATGCC3' and p53 antisense: 5'TCGAGTCTGTAGGATCTGTAGGATTGGCCAATCTGTACGGATCTGTACGGAATTCGA3') were annealed and introduced into the PCR2.1 TOPO vector (Lifetechnologies). The p53-responsive element was digested with KpnI/XhoI and introduced into the vector pGL4.10 [luc2] (Promega, Charbonnières-les-Bains, France) digested with the same enzymes.

### Extract library

One hundred and sixty-four methanolic extracts dissolved in dimethyl sulfoxide (DMSO) were obtained from the "Chimiothèque Nationale" (http://chimiotheque-nationale.cn.cnrs.fr/) and especially from the "Chimiothèque- Extractothèque" of the "Ensemble des Resources biologiques, Cellules vivantes et cryoconservées" of the collections of the Muséum national d'Histoire naturelle—(http://www.mnhn.fr/fr/collections/ensembles-collections/ressources-biologiques-cellules-vivantes-cryoconservees). The extracts were screened at 10 ng/μl in 96-well plates. Each plate also contained triplicate wells of negative control (1% DMSO only) and positive control (1 mg/ml G418 and 1% DMSO (vehicle)).

### H7 extract preparation

Eight kilograms of fresh *Lepista inversa* mushrooms, also identified as *Lepista flaccida*/*Paralepista flaccida*, *Paralepista inversa*, (common names *Clitocybe inversa*/*Clitocybe flaccida*), were collected between November 2014 and January 2015 from the areas of the “Bois de Vincennes” (48°49'40.95"N, 2°27'50.91"E) in the southeast suburb of Paris (Vincennes, Val-de-Marne, France), and of the “Parc du Sausset” (48°57'42.75"N, 2°30'18.08"E) in the northeast suburb of Paris (Villepinte, Seine-Saint-Denis, France). The collected mushrooms were identified thanks to the expertise of the French Mycological Society and ultimately by comparison with specimens of the MNHN herbarium. After being dusted off, the mushrooms were stored overnight at -80°C before freeze-drying at -110°C under a secondary vacuum for 24 to 48 h. Dried mushrooms (646 g) were crushed in a mortar and stored in glass pots at room temperature. The mushroom powder was extracted three times with a hydro-alcoholic solution composed of 50% distilled water (pH 6) and 50% distilled methanol (1 l per 10 g lyophilized mushroom powder). The three extracts were pooled and concentrated under reduced pressure (Rotavapor, Büchi), yielding 103 g crude extract, which was stored at -20°C.

### Screening assay

Lipofectamine LTX (ThermoFisher Scientific, Waltham, MA, USA) was used according to the manufacturer’s recommendations to transfect HeLa cells with each FLuc-int-PTC construct. The cells were distributed 24 h later into white 96-well plates (Corning, Corning, NY, USA) and incubated for 20 h at 37°C and 5% CO2 with extract at 10 ng/μl or with G418 at 1 μg/μl. Luciferase activity was then measured by adding the Steadylite plus luciferase substrate (Perkin Elmer, Waltham, MA, USA) to each well and by measuring the visible light from each well for 10 sec with a Tristar luminometer (Berthold technologies, Versailles, France). The plate was read twice and the reported values are averages of the two reads.

### Protein extraction

Proteins were extracted from 2x10^6^ cells in lysis buffer containing 5% SDS, 50 mM Tris-HCl pH 7, 20 mM EDTA, and a protease inhibitor cocktail (HALT protease inhibitor, Pierce-Biotechnology, Rockford, IL, USA). Lysates were subjected to 30 pulses of sonication (Branson Digital Sonifier/amplitude 20%) before a 5-min centrifugation at 8000 g. The supernatant was collected and used as protein extract.

### Western blotting

The equivalent of 2.5x10^5^ cells was subjected to 10% SDS-PAGE before transfer of the proteins to a nitrocellulose membrane. The membranes were incubated overnight at 4°C in the presence of a 1/200 dilution of anti-p53 antibody (DO1) (Santa Cruz Biotechnology, Dallas, TX, USA), a 1/20000 dilution of anti-Fluc antibody (Abcam, ab185924),or a 1/1000 dilution of the anti-CBP80 (H-300) antibody (Santa Cruz Biotechnology, Dallas, TX, USA). After three washes of the membrane in TBS Tween, the membranes were exposed to a solution of peroxidase-coupled secondary antibody for detection of mouse- or rabbit-raised antibody (Jackson ImmunoResearch, Suffolk, UK). Antibodies were then detected with Super Signal West Femto Maximum Sensitivity Substrate (Pierce-Biotechnology, Rockford, IL, USA).

### RT-PCR

The RT-PCR procedure and the primers used in this study have been described previously [42] except for the splicing analysis of the construct Fluc-int-WT (sense primer: 5'AGATCCTATTTTTGGCAA3'; antisense primer: 5'TGGCGACGTAATCCACGATC3') and the Rluc amplification (sense primer: 5'ATGACTTCGAAAGTTTATG3'; antisense primer 5'TTCAGATTTGATCAACGCA3').

### Cell population growth

5.0x10^4^ Calu-6 cells were plated on day 0 before adding DMSO, H7 extract at 25 ng/μl, or G418 at 1μg/μl. Every two days up to day 10, the cells were counted with a Tali cell cytometer (ThermoFisher Scientific, Waltham, MA, USA) and the medium with or without H7 or G418 was renewed.

### Inhibition of NMD by cycloheximide treatment

To inhibit NMD, cells were exposed to 200 μg/ml cycloheximide (USBiological, Swampscott, MA, USA) for 4 h before harvest. RNA was purified with RNazol (MRC, Cincinnati, OH, USA).

### Combined firefly and renilla luciferase activity measure

The measure of the firefly luciferase and renilla luciferase activities was performed using the Dual Luciferase Assay according to the technical protocol of the supplier (Promega; E1910).

### Measuring CFTR functionality on cystic fibrosis patient cells

The assay used to measure CFTR functioning was essentially as reported previously [60], except that patient cells were collected at the hospital by nose smear, kept in RPMI medium (Gibco, ThermoFisher Scientific, Waltham, MA, USA) at room temperature, and sent to the experimental laboratory for further experiments. The cells were centrifuged at 300 g for 5 min. before plating at about 5000 cells/well on 96-well black CellBind-treated plates (Corning, Corning, NY, USA). They were then incubated as previously reported for 24 h with 6-methoxy-*N*-(3-sulfopropyl)quinolinium (SPQ) (ThermoFisher Scientific, Waltham, MA, USA) and DMSO, H7, or G418 prior to incubation in 13.5 mM NaI for 30 min at 37°C and 5% CO_**2**_. The fluorescence was measured 8 times in 2 min, with a Tristar fluorimeter (Berthold technologies, Versailles, France). The cells were then incubated in 13.5 mM NaNO_**3**_ medium with CFTR activators (200 μM IBMX and 20 μM Forskolin) (ThermoFisher Scientific, Waltham, MA, USA) and the fluorescence was measured 40 times in 10 min. An increase of fluorescence reflects the replacement of iodide on the SPQ molecule by NO_**3**_^**-**^ linked to export of iodide from the cells by a functional CFTR.

All cystic fibrosis patient cells used in this study were obtained under the authorization of the local ethic committee "Comité de Protection des Personnes Nord Ouest IV". Patients were informed about the study and provided a written consent.

## Results

### Design of a new screening system for identifying effective correctors of nonsense mutations

**Fig 1:**
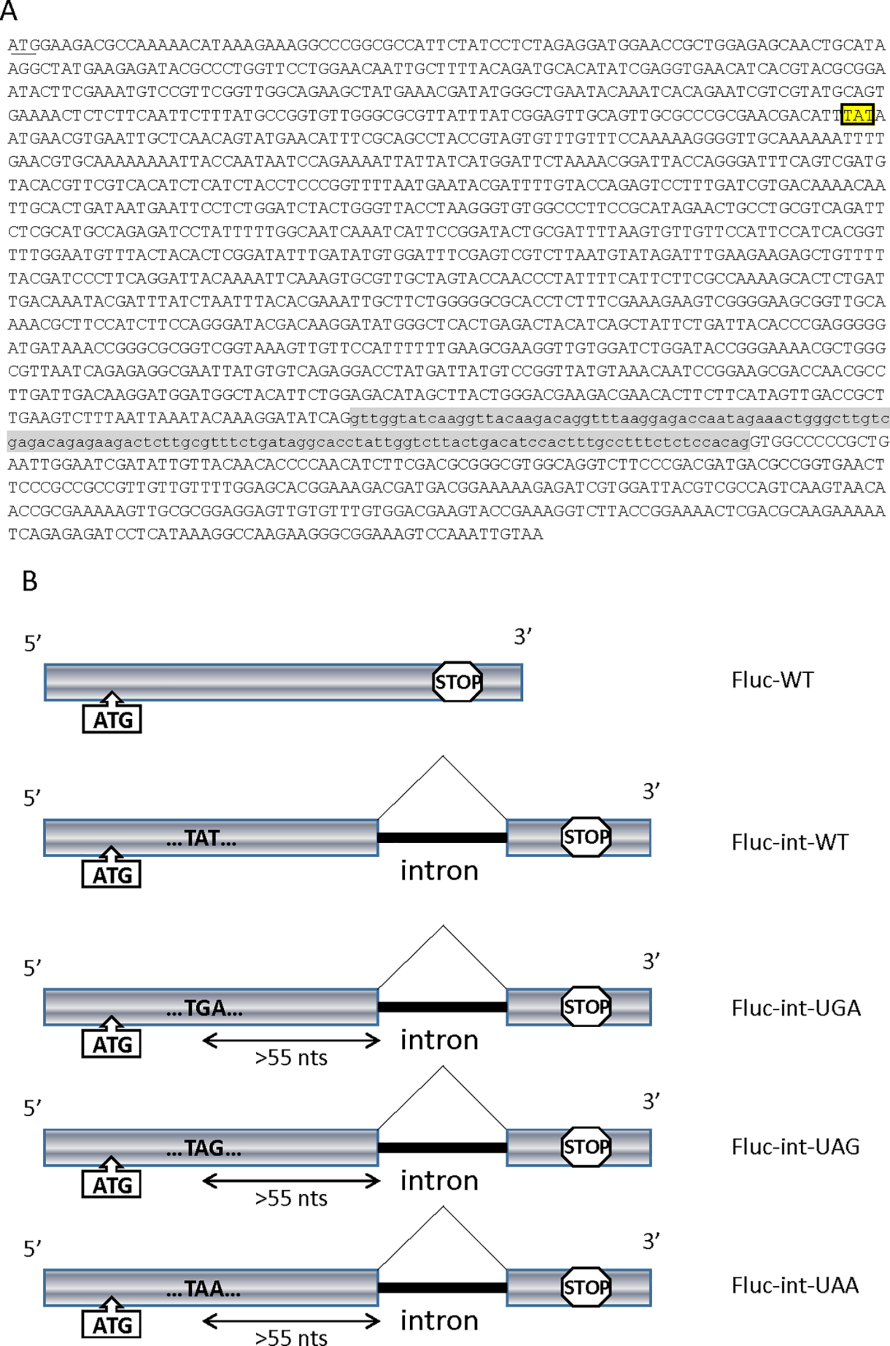
Schematic representation of constructs. (A) Coding sequence of firefly luciferase, used in this study. The initiation codon is underlined. The codon used to generate a nonsense mutation is framed. The intron sequence is in small letters and highlighted in gray. (B) Schematic representation of the firefly luciferase (Fluc) constructs: Fluc-WT is the original cDNA encoding the firefly luciferase; Fluc-int is the same cDNA with an added intron (horizontal thick black line), giving rise to a splicing-prone RNA with a wild-type open reading frame. Fluc-int-UGA, Fluc-int-UAG, and Fluc-int-UAA were derived from Fluc-int by introducing the DNA sequences corresponding to the premature termination codons UGA, UAG, and UAA, respectively.

**Fig 2:**
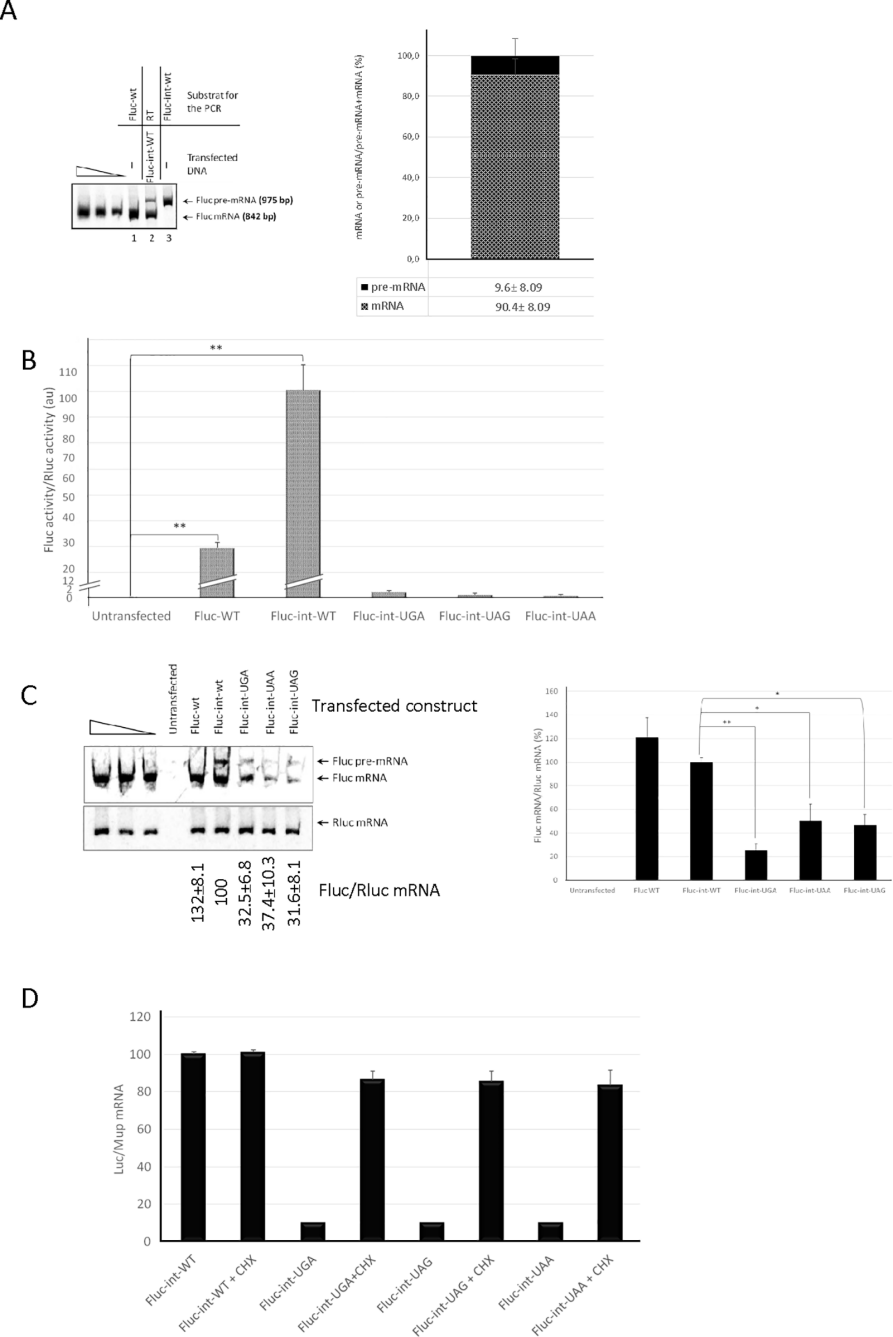
Validation of the reporter genes used in the screen. (A) The Fluc-int-WT reporter RNA is spliced. PCR amplification was performed on the Fluc-WT or Fluc-int-WT construct (lanes 1 and 3, respectively), or on the reverse-transcription reaction (RT) performed with extracted RNAs from HeLa cells transfected with the Fluc-int-WT construct (lane 2) in the presence of radioactive dCTP(α^33^P). The amplified fragments were electrophoresed through an acrylamide gel to demonstrate that the intron introduced in the firefly luciferase cDNA is efficiently spliced out. The position of each species is indicated on the right side of the gel and a quantification of the relative proportion of pre-mRNA (black box) and mRNA (spotted box) is shown on the right panel. (B) Luciferase expression associated with the constructs used in this study. Firefly luciferase activity normalized by renilla luciferase measured in wells of a 96-well plate containing untransfected HeLa cells, or HeLa cells transfected with pRluc and pFluc-WT, pFluc-int-WT, pFluc-int-UGA, pFluc-int-UAG, or pFluc-int-UAA constructs. (C) Measure of the firefly luciferase (Fluc) and renilla luciferase (Rluc) mRNAs by RT-PCR from HeLa cells transfected with pRluc and pFluc-WT, Fluc-int-WT, Fluc-int-UGA, Fluc-int-UAG, or Fluc-int-UAA constructs. (D) The extent of NMD is shown on a bar plot depicting levels of Fluc-int-PTC RNAs measured by quantitative RT-PCR in the absence and in the presence of cycloheximide (+CHX). The values shown are from two independent experiments. Error bar = S.D., Student t-test: **P<0.01; ***P<0.001.

To identify a molecule capable of correcting a nonsense mutation in human cells, selection should be done on a spliced mRNA harboring a nonsense mutation in order to take most gene expression pathways into account. For this, an intron was introduced into the second half of the cDNA encoding firefly luciferase (Fluc), between codons 448 and 449 (at nucleotide 1344) to generate the Fluc-int-WT construct (Fig 1). After transient transfection of HeLa cells with the pFLuc-int-WT plasmid for 48 hours (Fig 1B), RNAs were purified and reverse-transcribed in order to monitor the splicing efficiency after PCR amplification with primers surrounding the intron position (Fig 2A). The results show that the Fluc-int-WT DNA construct results in a predominantly spliced mRNA after transfection in HeLa cells since about 90% of Fluc RNA is mRNA, indicating that the spliceosome can recognize the artificial intron and efficiently splice it out. It is worth noting that the splicing event did not impair the function of the luciferase enzyme, since luciferase activity was detected upon expression of the Fluc-int-WT construct (Fig 2B). Actually, the splicing event improved synthesis of functional firefly luciferase, since the level of firefly luciferase activity measured in the presence of the Fluc-int-WT construct and normalized for transfection efficiency by renilla luciferase activity was more than 4 times that detected with the firefly luciferase cDNA (construct Fluc-WT, encoding a splicing-immune mRNA) (Fig 2B). Higher protein synthesis from spliced mRNAs as compared to mRNAs from intron-less pre-mRNAs has been reported in several studies [61,62]. In addition, the difference of firefly luciferase activity between the two constructs cannot be related with a difference in the transcription rate since their transcription level is similar (Fig 2C).

Next, a nonsense codon was introduced into the Fluc-int-WT construct at codon 109, leaving a distance of 1017 nucleotides between the PTC and the intron (Fig 1). According to the “50–55 nucleotides rule” [63], introduction of a PTC at this position in the Fluc-int-WT construct should induce NMD of the corresponding Fluc mRNA. To test this assumption, we measured the levels of Fluc-int-WT and Fluc-int-PTC RNAs (Fluc-int-UGA, -UAG, and -UAA RNAs) normalized by the level of the exogenous MUP mRNA in the presence and absence of the well-known NMD inhibitor cycloheximide (CHX) (Fig 2D). As predicted, the level of each Fluc-int-PTC RNA was about 8-fold higher in the presence than in the absence of the NMD inhibitor, while the level of Fluc-int-WT RNA remained stable validating the NMD substrate status of Fluc-int-PTC RNAs.

Fluc-int-PTC constructs could thus be used to identify correctors of one, two, or all three nonsense codons by measuring the luciferase activity produced by cells expressing these constructs in the presence of the compounds to be tested. In the assay, above-background luciferase activity is observed only if a compound can inhibit NMD, activate readthrough, or both. Detection of luciferase activity means that in human cells the compound successfully corrects the nonsense mutation present on a construct encoding a splicing-prone RNA. HeLa cells were transiently and separately transfected with each of the three Fluc-int-PTC constructs (Fig 1B), equally distributed in 96-well plates, and incubated with DMSO or with a compound to be tested. The test compounds included, as positive control, the compound G418, known to inhibit NMD and activate readthrough substantially at the concentration used (1 mg/ml) [51,64,65,66] (S1 Fig). To select very effective correctors of nonsense mutations, only compounds inducing a luciferase activity higher than that measured in the presence of G418 were considered.

### Identification of a *Lepista inversa* extract as a powerful corrector of nonsense mutations

**Fig 3:**
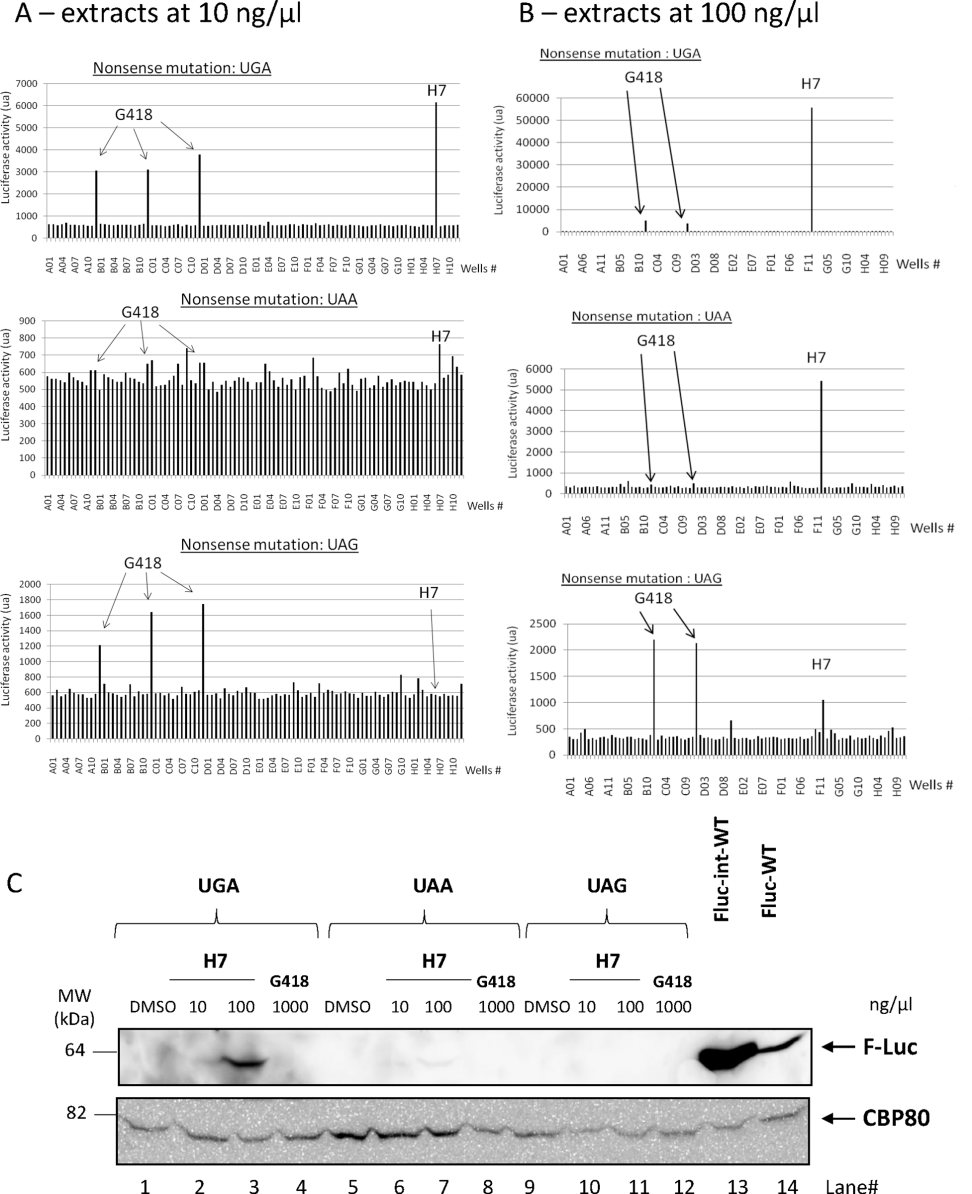
Identification of the H7 extract by screening. Fungal and marine invertebrate extracts at 10 ng/μl (A) or at 100 ng/μl (B) were tested for their capacity to induce luciferase activity from constructs Fluc-int-UGA (upper panels), Fluc-int-UAA (middle panels), and Fluc-int-UAG (lower panels). (A) The extract in well H7 promoted high luciferase activity reflecting good correction of the UGA or UAA nonsense mutation. G418 at 1000 ng/μl in wells B1, C1, and D1 was used as positive control and served to distinguish strong nonsense mutation correctors (luciferase activity higher than that induced by G418) from weaker ones (luciferase activity equal to or lower than that induced by G418). (B) H7 at 100 ng/μl was introduced in well G1 and G418 at 1000 ng/μl in wells C1 and D1. (C) Western-blot analysis to detect the presence of the firefly luciferase (F-Luc) protein after transfection of HeLa cells with pFluc-int-UGA (lanes 1–4), pFluc-int-UAA (lanes 5–8), pFluc-int-UAG (lanes 9–12), pFluc-int-WT (lane 13) or pFluc-WT (lane 14). CBP80 was used as a loading control.

A library composed of 164 extracts of fungi and marine invertebrates was constituted. Each extract was tested at 10 ng/μl for its capacity to correct the nonsense mutations of the three Fluc-int-PTC constructs (Fig 3A). When cells expressing the Fluc-int-UGA construct were used, well H7 showed about twice as much activity as the wells containing G418. The corresponding extract also induced higher luciferase activity than G418 in cells transfected with the Fluc-int-UAA construct, but the activity was very close to the background level at the extract concentration used. This result suggests that the H7 extract corrects the UAA nonsense mutation less effectively than the UGA nonsense mutation. At the concentration used in the screening assay, the H7 extract was unable to correct the UAG nonsense mutation, in contrast to G418. The results presented in Fig 3A thus show that the extract added at position H7 (henceforth called H7) corrects the UGA and UAA nonsense mutations more effectively than G418 (at 1 mg/ml) but cannot correct the UAG nonsense mutation.

H7 originated from the mushroom *Lepista inversa*, a common edible mushroom. To see whether a higher concentration of H7 might correct the UAG nonsense mutation, H7 was tested at 100 ng/μl (Fig 3B). At this concentration, H7 proved able to correct the UGA nonsense mutation about 16 times more effectively and the UAA nonsense mutation almost 10 times more effectively than G418. It was also able to rescue expression of the UAG-harboring firefly luciferase gene, but less effectively than G418 (Fig 3B).

To correlate the luciferase activity with the expression from the construct Fluc-int-PTC upon H7 or G418 treatment, HeLa cells were transfected with pFluc-int-UGA, pFluc-int-UAA or pFluc-int-UAG and treated with DMSO, H7 or G418 for 24 hours before analyzing the firefly luciferase protein level by western-blotting (Fig 3C). The firefly luciferase protein was only detected from the expression of pFluc-int-UGA or pFluc-int-UAA upon 100 ng/μl H7 treatment and barely upon 10 ng/μl H7 treatment. G418 was able to promote the expression of a detectable level of firefly luciferase protein from the expression of pFluc-int-UGA construct only, demonstrating that western-blotting is less sensitive than the measure of the firefly luciferase activity (Fig 3A and 3B). Overall, the results presented in Fig 3 demonstrate that H7 extract corrects UGA and, to a lesser extent, UAA nonsense mutations but not UAG nonsense mutations.

### H7 corrects endogenous nonsense mutations, allowing synthesis of a functional protein

**Fig 4:**
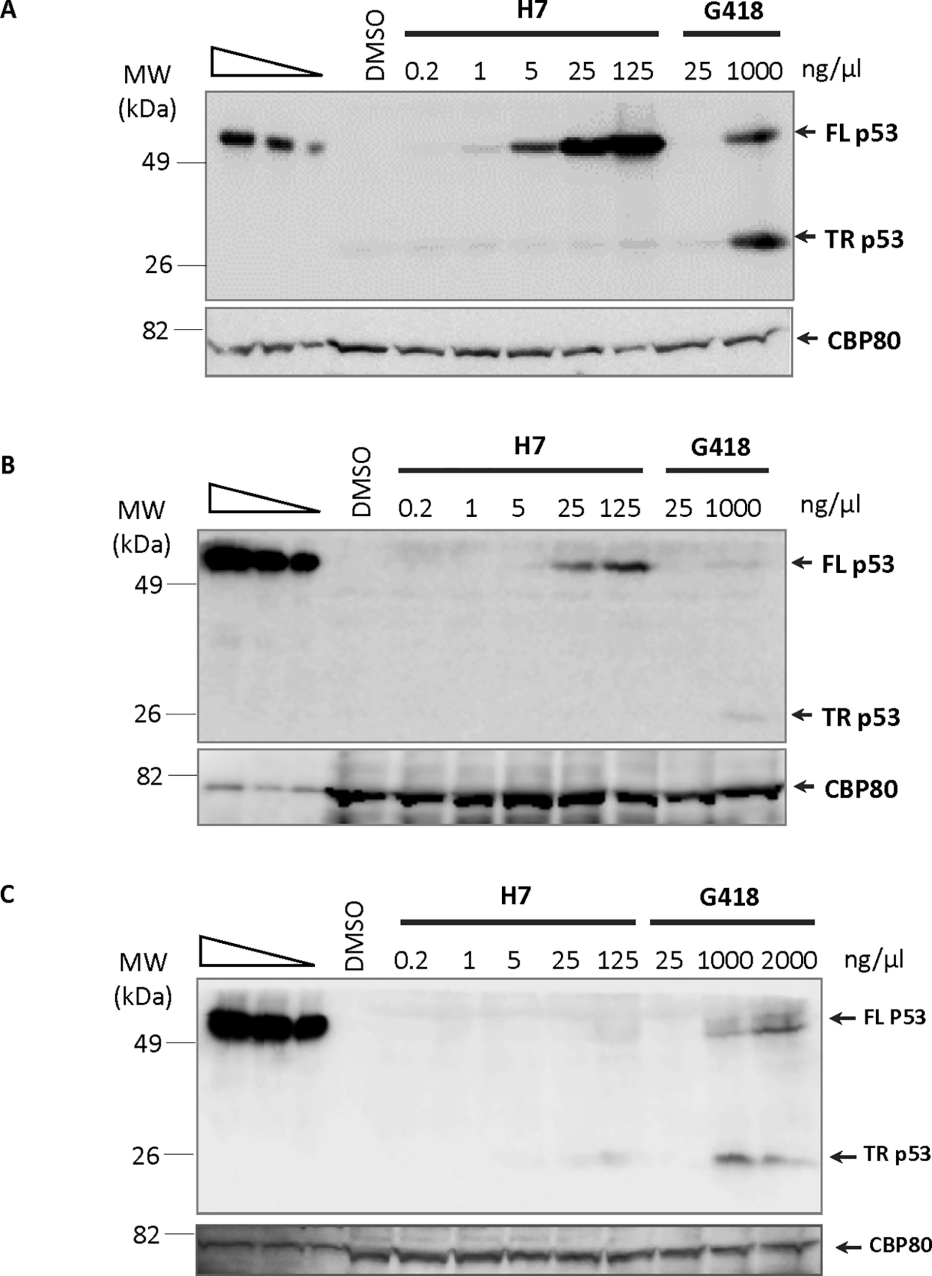
H7 restores expression of the *TP53* gene harboring a UGA or UAA nonsense mutation but not of the *TP53* gene harboring a UAG mutation. (A) Calu-6 (UGA nonsense mutation at codon 196 of the *TP53* gene), (B) Caov-3 (UAA nonsense mutation at codon 136 of the *TP53* gene), or (C) Caco-2 (UAG nonsense mutation at the codon 204 of *TP53* gene) cells were incubated for 24 h with DMSO as control or with H7 in increasing amounts (from 0.2 to 125 ng/μl) or G418 (from 25 to 1000 ng/μl for (A) and (B) or from 25 to 2000 ng/μl for (C)) before protein extraction and analysis. Western blotting with anti-p53 antibody raised against the N-terminal part of the protein or with anti-CBP80 antibody as a loading control. Truncated (p53 TR) and full-length p53 (p53 FL) are indicated on the right side of each gel and the molecular weight (MW) is shown on the left side of each gel. The three leftmost lanes show twofold serial dilutions of untreated HeLa cell extract. The results presented in Figure are representative of three independent experiments.

To validate the capacity of H7 to correct UGA and UAA nonsense mutations, cell lines harboring different nonsense mutations in the *TP53* gene were incubated with H7 before measuring the level of p53 protein by western blotting (Fig 4). Consistently with the screening results (Fig 3), H7 corrected the UGA nonsense mutation very effectively, since full-length p53 was detected with an H7 extract concentration as low as 0.2 ng/μl (Fig 4A). Correction of the UAA nonsense mutation required a higher concentration of H7 (at least 5 ng/μl) (Fig 4B). Even at the highest H7 concentration used (125 ng/μl), correction of the UAG nonsense mutation was barely observed (Fig 4C). The results presented in Fig 4 are thus in very good agreement with the screening data. They confirm the ability of H7 to correct the UGA and UAA nonsense mutations but not (or very poorly) the UAG nonsense mutation.

Interestingly, the truncated p53 protein corresponding to translation from the translation initiation codon to the PTC was observed with the anti-p53 antibody raised against the N-terminal part of the protein (Fig 4). In all three cell lines, the level of truncated p53 protein was found to increase in the presence of G418. This is consistent with the capacity of G418 to inhibit NMD [66]. With H7, in contrast, only full-length p53 was found to increase. This suggests that H7 is unable to inhibit NMD (see below).

### H7 does not inhibit NMD

**Fig 5:**
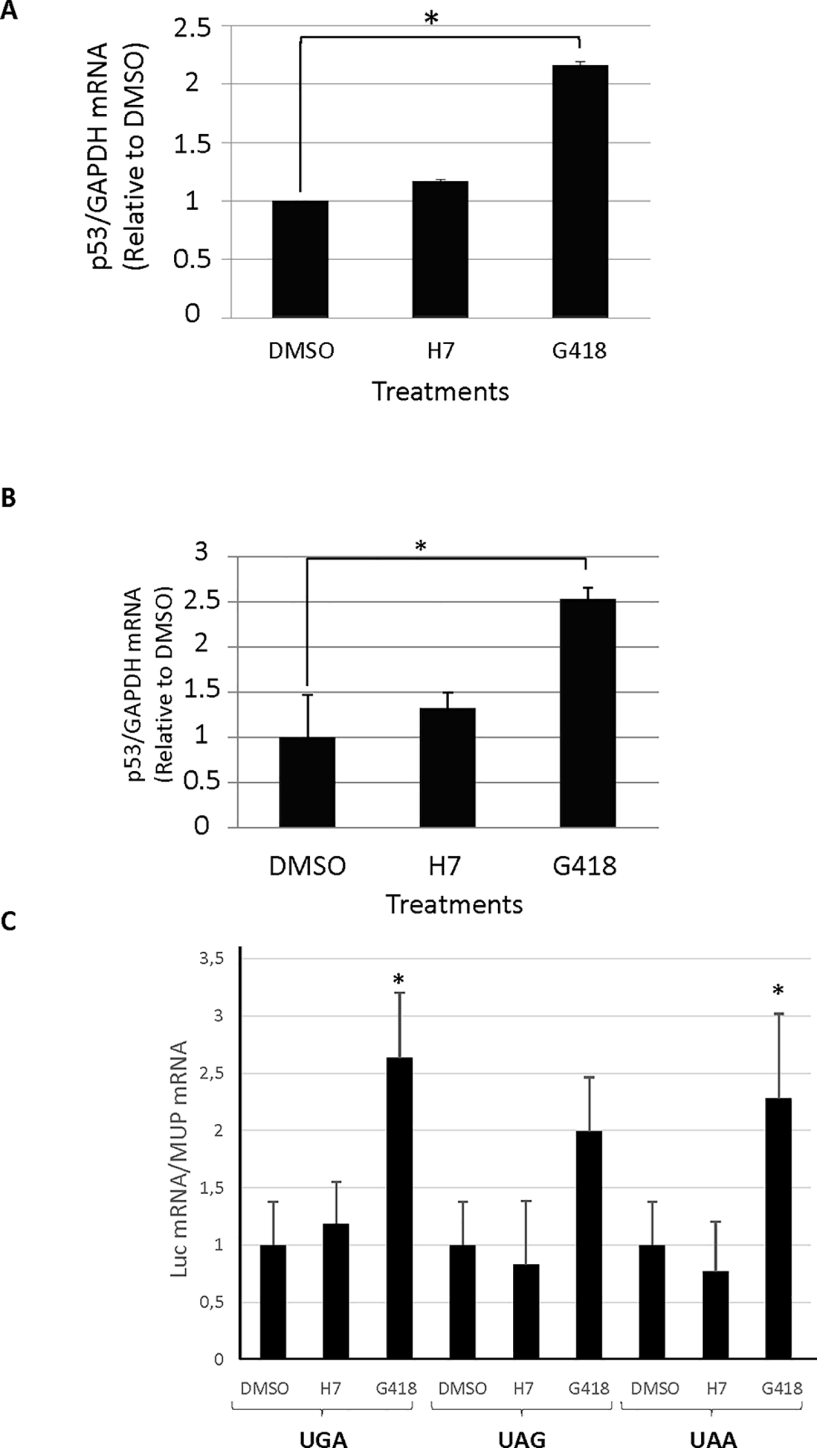
H7 is not an NMD inhibitor. Calu-6 (A), Caov-3 (B) cells and HeLa cells transfected with pFluc-int-PTC and pIE-MUP (C) were incubated with DMSO, H7 extract at 25 ng/μl, or G418 at 1000 ng/μl for 24 h before RNA extraction and RT-qPCR. Quantification based on two independent experiments is shown. Error bar = S.D., Student t-test: *P<0.05.

To test the ability of H7 to inhibit NMD, Calu-6 and Caov-3 cells were incubated for 24 h with H7 or G418 before RNA extraction and measurement of the level of p53 mRNA (Fig 5A and 5B). As expected, the level of p53 mRNA was about twice as high in the presence of G418 as in the controls, consistently with the capacity of G418 to inhibit NMD [42,66]. H7 treatment, in contrast, did not significantly change the level of p53 mRNA, indicating that H7 is not an inhibitor of NMD. These results are consistent with those of the western blot analysis (Fig 4) showing a higher level of full-length but not truncated p53 protein in the presence of H7, but higher levels of both forms in the presence of G418. The effect of H7 treatment on NMD was also assessed by measuring the level of Fluc-int-PTC mRNAs normalized by the MUP mRNA level. Unlike G418, H7 did not significantly change the level of Fluc-int-PTC mRNAs, indicating that H7 is not an inhibitor of NMD (Fig 5C).

### Determination of H7 toxicity

**Fig 6:**
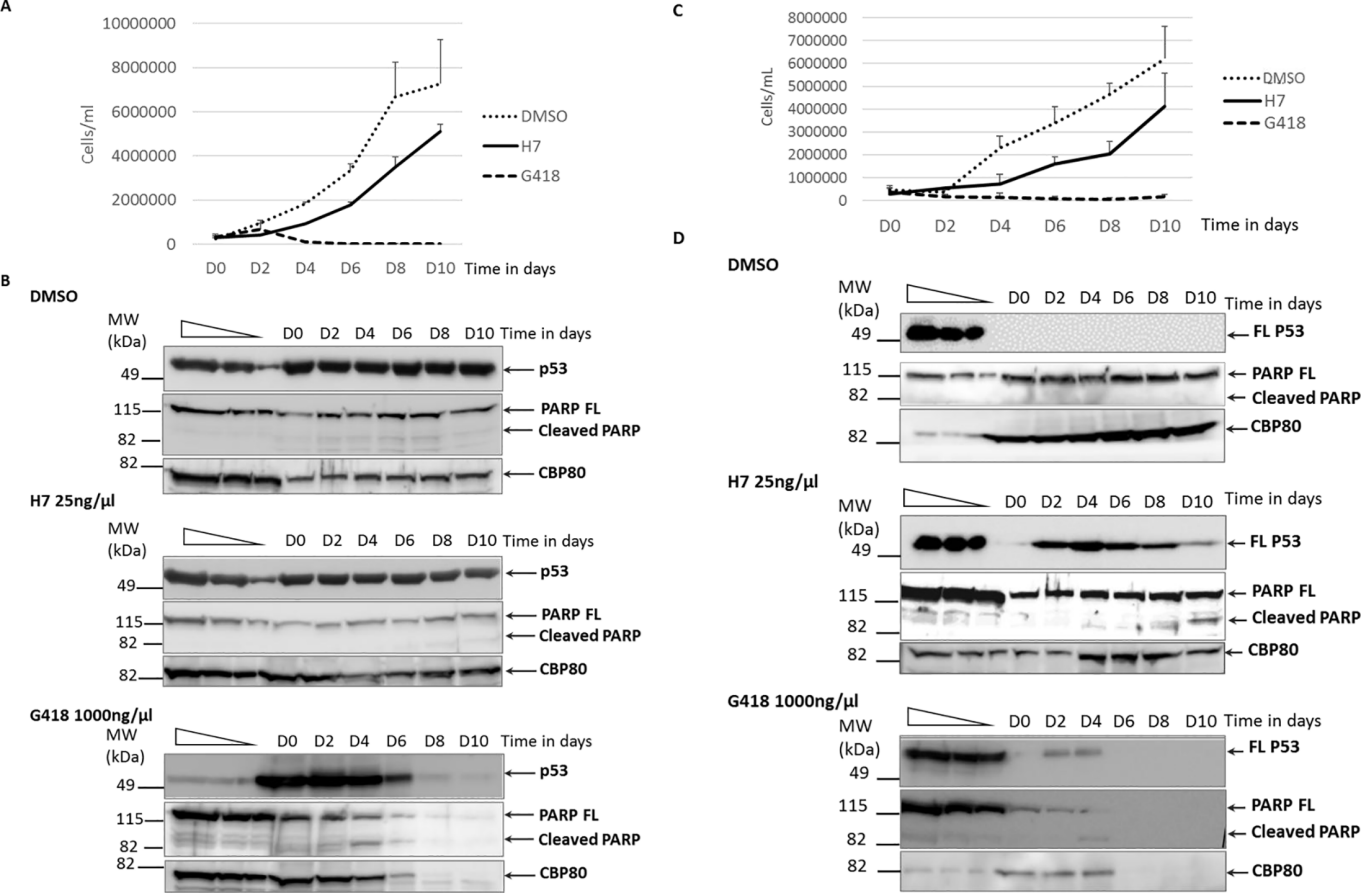
Cell toxicity in the presence of DMSO, H7, or G418. Cell proliferation was measured in the presence of DMSO, H7 extract, or G418. Calu-6 cells were counted and treated with DMSO (dashed line), H7 extract (H7) at 25 ng/μl (black line) or G418 at 1000 ng/μl (dotted line) every two days from day 0 (D0) to day 10 (D10). Error bars represent standard deviations. The results presented in the figure are based on two independent experiments.

The toxicity of H7 was assessed by measuring the impact of the extract on the growth of 16HBE14o- cells, which are immortalized lung cells from a healthy person [67]. The cells were treated with H7, DMSO, or G418 for 10 days (Fig 6A), with renewal of the culture medium and treatment every two days. Quickly after the start of cell incubation with G418, growth slowed down and all the cells eventually died. Cells treated with H7 grew more slowly than DMSO-treated cells during the first four days of treatment, but growth then accelerated to reach a rate similar to that of control cells. H7 thus appears much less toxic than G418. These results are supported by the observation that cleaved PARP, a marker of apoptosis, was detected in G418-treated cells from day 4 of treatment but not at any time in DMSO- or H7-treated cells (Fig 6B).

To see if H7 treatment remained effective for 10 days, the same experiment was done with Calu-6 cells, in which correction of a UGA nonsense mutation in p53 can be monitored. Like the 16HBE14o- cells, the Calu-6 cells showed slower growth over the first four days of H7 treatment, but growth similar to that of DMSO-treated cells thereafter. The G418-treated Calu-6 cells, like the similarly treated 16HBE14o- cells, died off completely within 4 days (Fig 6C). As expected, Calu-6 cells treated with DMSO did not synthesize full-length p53 protein at any time in the course of the experiment, unlike cells exposed to H7 extract, which synthesized full-length p53 protein throughout the exposure period (Fig 6D). The presence of p53 in cells treated with H7 extract demonstrates that H7 treatment was active over the ten-day period. A slight decrease in the level of p53 was observed from day four, corresponding with limited activation of apoptosis, as shown by the appearance of cleaved PARP protein. This limited apoptosis might be the consequence of resumed p53 synthesis, as previously reported in these cells [68].

### H7 can correct nonsense mutations in cell lines and cells from cystic fibrosis patients by promoting the synthesis of functional proteins

**Fig 7:**
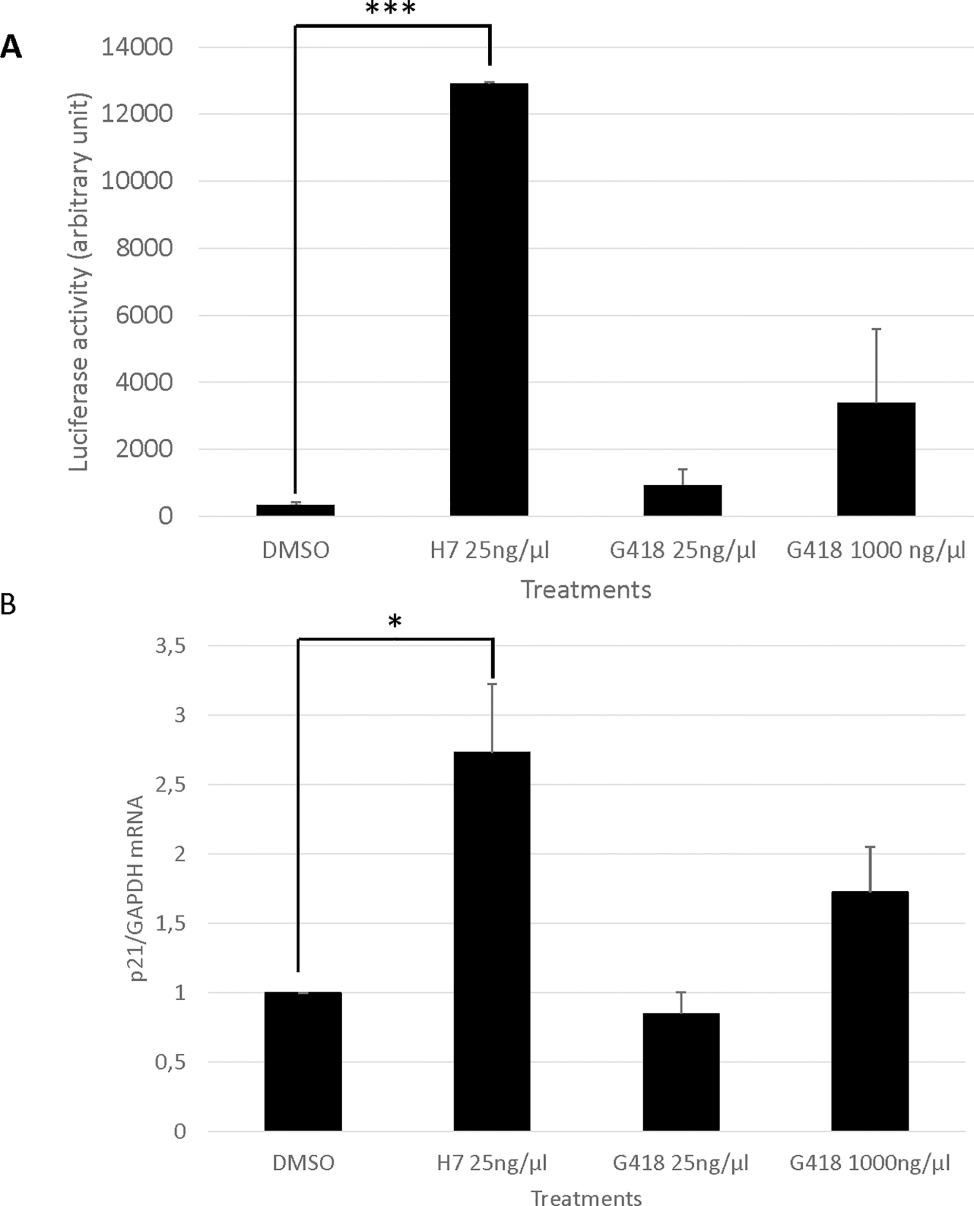
H7 extract promotes the synthesis of functional p53 protein in Calu-6 cells. (A) Calu-6 cells were transfected or not (untransfected) with an expression vector carrying a luciferase gene under the transcriptional control of a p53 response element. The cells were then incubated with DMSO, H7, or G418. Luciferase activity in the cells was measured 24 h after the start of treatment. Activities are expressed in arbitrary units. (B) The level of p21 mRNA was measured in Calu-6 cells exposed to DMSO, H7, or G418. The results presented in the figure are representative of three independent experiments. Error bar = S.D., Student t-test: *P<0.05; ***P<0.001.

If correction of a nonsense mutation is to be used therapeutically, synthesis of a full-length protein is not enough. The function of the normal protein must also be preserved. The next step was therefore to assess rescue of the functions of PTC-containing genes. P53 is a transcription factor with a panel of clearly identified targets (e.g. p21) [69,70]. To quantify rescue of p53 function in Calu-6 cells, constructs expressing the firefly luciferase gene under the transcriptional control of a p53-responsive element in the promoter were introduced into cells by transient transfection. The cells were then incubated with DMSO, H7, or G418 for 24 h before measuring the luciferase activity (Fig 7A). In this experiment, the luciferase activity was about 40 times as high in H7-treated as in DMSO-treated cells, suggesting that the p53 protein synthesized upon H7 treatment is functional. Consistently with the results shown in Fig 4, treatment with G418 at 25 or 1000 ng/μl was also found to enhance luciferase activity, but to a lesser extent (about 10-fold).

Another way to determine whether the p53 protein synthesized upon H7 or G418 treatment is functional in Calu-6 cells is to measure the level an endogenous transcriptional target of p53 such as p21 (Fig 7B). The level of p21 mRNA was thus measured in H7-, G418-, and DMSO-treated cells. As compared to DMSO treatment, H7 treatment was found to cause a 2.5- to 3-fold increase in p21 gene mRNA. The increase observed with G418 treatment was only 1.7- to 2-fold at the highest tested G418 concentration. This means that H7 can at least rescue the function of a *TP53*-PTC-containing gene in Calu-6 cells. Surprisingly G418 treatment, which induces synthesis of full-length p53 protein in Calu-6 cells at a significant level (Fig 4A), was unable to promote high luciferase activity (Fig 7A) or p21 mRNA (Fig 7B). This indicates that the p53 synthesized upon G418 treatment is not functional, probably because an inappropriate amino acid is incorporated at the PTC position. Alternatively, the high level of truncated p53 protein synthesized in the presence of G418 might interfere with the activity of the full-length p53 protein.

**Fig 8:**
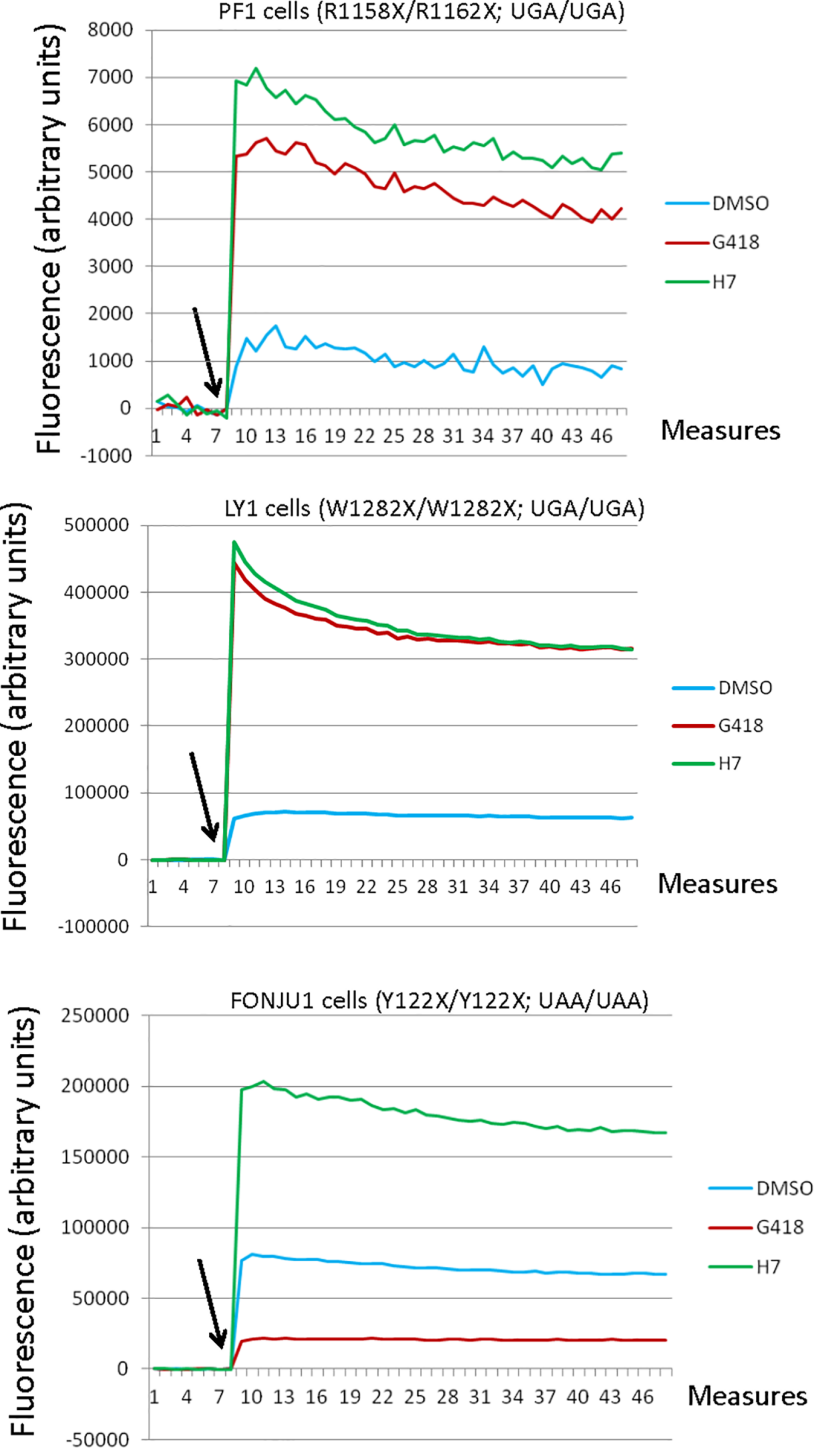
H7 extract promotes the synthesis of functional CFTR in cystic fibrosis patient cells. Patient cells harboring a nonsense mutation on each allele of the CFTR gene were treated with DMSO (blue line), H7 (green line), or G418 (red line) before measuring CFTR-mediated transmembrane ion transport (iodide efflux). The fluorescence intensity, due to the halide-sensitive dye SPQ, depends on the quantity of functional CFTR protein at the cell membrane. The arrow indicates the moment when a cAMP-stimulating cocktail was added to the cells. The upper panel corresponds to R1158X (UGA)/R1162X (UGA) patient cells (PF1 cells). The middle panel corresponds to W1282X (UGA) /W1282X (UGA) patient cells (LY1 cells). The lower panel corresponds to Y122X (UAA)/Y122X (UAA) patient cells (FONJU1 cells).

To further assess the functional efficacy of H7, we sought to correct PTCs in primary cells from cystic fibrosis patients harboring a nonsense mutation in the cystic fibrosis transmembrane conductance regulator gene (CFTR). These patients were selected from the French National Cystic Fibrosis Register as having a nonsense mutation on both CFTR alleles. It was not required that the same position be mutated on both CFTR alleles, as long as each allele bore a nonsense mutation. The selected cells carried the following nonsense mutations in the CFTR gene: R1158X (UGA)/R1162X (UGA) (PF1 cells), W1282X (UGA)/W1282X (UGA) (LY1 cells), or Y122X (UAA)/Y122X (UAA) (Fonju1 cells). The cells were first incubated for 20 h with the halide-sensitive fluorescent indicator 6-methoxy-N-(3-sulfopropyl)quinolinium (SPQ) and DMSO, G418, or H7, before measurement of iodide export through the cell membrane, which depends on the amount of functional CFTR present at the cell membrane [60] (Fig 8). The results in Fig 8 show that H7 extract was able to restore the function of CFTR in cells from all three patients. In PF1 and Fonju1 cells, H7 appeared as a better nonsense mutation corrector than G418, as it gave rise to a higher fluorescence level. The difference between the two treatments was greatest in patient cells with the UAA nonsense mutation. This result is consistent with those of the screen (Fig 3), showing correction of UAA by H7 but not by 1000 ng/μl G418.

## Discussion

Nonsense mutations are reported to cause about 10% of genetic disease cases [17]. To counteract the presence of a nonsense mutation in a gene, potential therapeutic approaches based on NMD inhibition and PTC readthrough have been developed [16,21,22]. Molecules with the capacity to correct nonsense mutations have been identified by screening. The screens used have often exploited constructs with a stop codon between two cDNAs or constructs bearing a cDNA encoding a protein with measurable activity (such as firefly luciferase) but for the presence of a nonsense mutation in the open reading frame [34,37,51,71]. Although these screening strategies target molecules with readthrough-promoting activity, they might not be appropriate for selecting clinically useful nonsense mutation correctors because they do not take NMD into account. NMD targets RNAs bearing both a PTC and traces of a splicing event. As more than 95% of human genes are transcribed to pre-mRNAs that undergo splicing before generation of mRNAs to be translated to proteins [52,53,54], NMD is liable to reduce considerably the level of a readthrough substrate of interest (up to less than 5% of the level of the corresponding wild-type mRNA) [24]. Hence, molecules capable of effectively rescuing expression of a gene harboring a nonsense mutation must either be able both to inhibit NMD and to activate readthrough [23,42,66,72] or be very strong activators of PTC readthrough. The novel screening system reported in this study is based on constructs expressing a pre-mRNA subject to splicing, making the mRNA sensitive to NMD when it harbors a PTC (Figs 1 and 2). The system is therefore particularly adequate for identifying molecules capable of correcting a nonsense mutation in human genes. We have demonstrated the validity of this system, using the known reference readthrough molecule G418. Having thus provided proof of concept, we have used the system to screen a library of natural extracts, to test its ability to reveal novel molecules with readthrough-promoting activity. We chose to select only compounds with higher correction efficacy than G418, taken as a reference readthrough-promoting molecule, in order to identify highly effective correctors of nonsense mutations.

Of the extracts tested, one (named H7) was selected for further study (Fig 3). H7 appears to correct the UGA and UAA nonsense codons very effectively and the UAG stop codon poorly or not at all (Figs 3 and 4). This is interesting because, on the basis of previous studies using aminoglycosides [27,51,73], UGA has been ranked as the most permissive stop codon and UAA as the least permissive. In other words, it was thought that the UGA stop codon is most easily read through and that readthrough of UAA is the most difficult. In contrast, the results obtained here with H7 point to UAG as the codon least permissive to readthrough. It thus appears that the readthrough efficiency depends both on the nucleotide context and on the molecule used to induce readthrough. It would seem that H7 and aminoglycosides induce readthrough via different modes of action, worthy of future investigation.

One way to improve readthrough efficiency is to inhibit NMD, as previously reported [57,58]. We show here that although H7 is a very effective corrector of nonsense mutations, it is not an NMD inhibitor (Fig 5). This suggests that H7 can induce the synthesis of abundant full-length protein from the residual population of PTC-containing mRNAs. This also suggests that the readthrough efficiency of H7 could be increased in the presence of NMD inhibitors [74,75,76] or readthrough potentiators such as CDX5-1 [77].

In immortalized cultured cells and in primary cells from cystic fibrosis patients, we have further shown the readthrough proteins synthesized upon H7 treatment to be functional (Figs 7 and 8). The higher efficacy observed with H7 than with G418 is consistent with the screening results and validates the working hypothesis that one should use a spliced PTC-mRNA to look for strong correctors of nonsense mutations in human cells.

The H7 extract originates from *Lepista inversa*, a common edible mushroom. At the concentration used to induce readthrough in our assays, this extract does not appear to be toxic (Fig 6). Like other mushrooms such as *Leucopaxillus giganteus* [78], *Lepista inversa* contains an exocyclic amino nucleoside called clitocine [79], known to rescue the expression of genes carrying a nonsense mutation more effectively than G418 [80], consistently with the data presented in this study. The converging results arising from previous studies on clitocine [68,80,81] and from our present study on *Lepista inversa* extract validate the screening system described here as adequate for identifying molecules with a high potential to correct nonsense mutations in human cells. This screening system provides a fast way to screen thousands of molecules. It is compatible with a high-throughput screening format and shows a low, homogeneous background level that makes it very easy to analyze the results and select positive molecules. Molecules selected with this screening system might be candidate drugs for the treatment of nonsense-mutation-related human pathologies.

The authors have declared that no competing interests exist.

## Supporting information

S1 Fig Correction of a UGA nonsense mutation by increasing amounts of G418. HeLa cells were transfected with the Fluc-int-UGA construct before exposure to increasing amounts of G418 for 24 h. Luciferase activity was then measured. The results of the figure are based on three independent experiments.
(TIF)
