# Supplementary figures and images for: Optimized approach for the identification of highly efficient correctors of nonsense mutations in human diseases
Source: PLoS One. 2017 Nov 13;12(11):e0187930. doi: 10.1371/journal.pone.0187930 (PMC5683606; doi:10.1371/journal.pone.0187930)

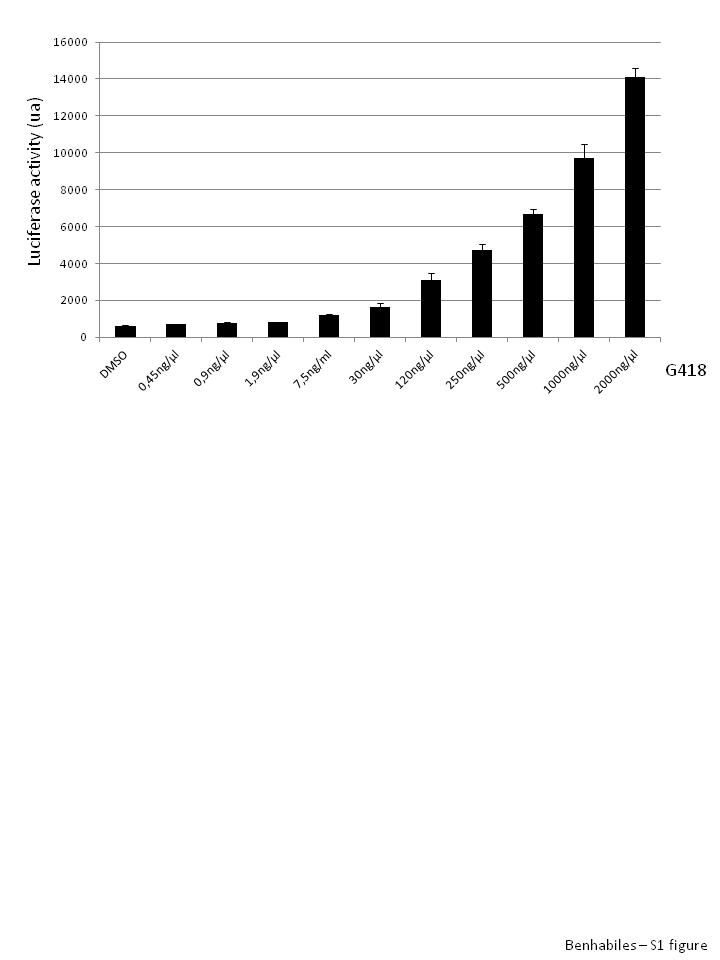

Supplement: S1 Fig — HeLa cells were transfected with the Fluc-int-UGA construct before exposure to increasing amounts of G418 for 24 h. Luciferase activity was then measured. The results of the figure are based on three independent experiments. (TIF) [file pone.0187930.s001.TIF]
